# Simplified control of neuromuscular stimulation systems for restoration of reach with limb stiffness as a modifiable degree of freedom

**DOI:** 10.1088/1741-2552/adc9e3

**Published:** 2025-05-06

**Authors:** Tyler R Johnson, Chase A Haddix, A Bolu Ajiboye, Dawn M Taylor

**Affiliations:** 1 Department of Neurosciences, Lerner Research Institute, The Cleveland Clinic, Cleveland, OH 44195, United States of America; 2 Department of Biomedical Engineering, Case Western Reserve University, Cleveland, OH 44106, United States of America; 3 School of Medicine, Case Western Reserve University, Cleveland, OH 44106, United States of America; 4 FES Center of Excellence, Louis Stokes Cleveland Department of Veterans Affairs Medical Center, Cleveland, OH 44106, United States of America

**Keywords:** functional electrical stimulation (FES), neuromuscular stimulation, arm movement, spinal cord injury, cocontraction, fatigue, brain–machine interfaces (BMI)

## Abstract

*Objective.* Brain-controlled functional electrical stimulation (FES) of the upper limb has been used to restore arm function to paralyzed individuals in the lab. Able-bodied individuals naturally modulate limb stiffness throughout movements and in anticipation of perturbations. Our goal is to develop, via simulation, a framework for incorporating stiffness modulation into the currently-used ‘lookup-table-based’ FES control systems while addressing several practical issues: (1) optimizing stimulation across muscles with overlap in function, (2) coordinating stimulation across joints, and (3) minimizing errors due to fatigue. Our calibration process also needs to account for when current spread causes additional muscles to become activated. *Approach.* We developed an analytical framework for building a lookup-table-based FES controller and simulated the clinical process of calibrating and using the arm. A computational biomechanical model of a human paralyzed arm responding to stimulation was used for simulations with six muscles controlling the shoulder and elbow in the horizontal plane. Both joints had multiple muscles with overlapping functional effects, as well as biarticular muscles to reflect complex interactions between joints. Performance metrics were collected *in silico,* and real-time use was demonstrated with a Rhesus macaque using its cortical signals to control the computational arm model in real time. *Main results.* By explicitly including stiffness as a definable degree of freedom in the lookup table, our analytical approach was able to achieve all our performance criteria. While using more empirical data during controller parameterization produced more accurate lookup tables, interpolation between sparsely sampled points (e.g. 20° angular intervals) still produced good results with median endpoint position errors of less than 1 cm—a range that should be easy to correct for with real-time visual feedback. *Significance.* Our simplified process for generating an effective FES controller now makes translating upper limb FES systems into mainstream clinical practice closer to reality.

## 1. Introduction

### 1.1. Functional electrical stimulation (FES) control strategies

FES (also called neuromuscular stimulation) has been used for decades to restore limb movements after paralysis by reanimating paralyzed muscles [1]. FES applied to the lower limbs and trunk has been used to restore standing, walking, and improve seated posture in individuals with tetraplegia [2–4]. FES for restoring basic hand function has been used by people with lower cervical spinal cord injuries (C5–C6) since the 90’s, and portable take-home FES hand systems have improved the ability of these paralyzed individuals to independently perform activities of daily living [5].

For people with injuries at the first through fourth cervical level of the spinal cord (C1–C4), not only does the FES system need to restore hand grasp and orientation, but also hand placement in space. Control of these additional shoulder and elbow degrees of freedom requires coordinated activation of multiple muscles impacting both the shoulder and elbow joints.

Many different approaches to controlling the arm with FES have been explored. Different FES controller options have been developed and demonstrated using computational biomechanical models [6–14], able-bodied [11, 15–18] and paralyzed [13, 19] humans, and intact non-human primates where paralysis was modelled by general anesthesia [20] or focal nerve block [21–23]. Not only have biomechanical models been used to simulate and test FES controllers, they have also been incorporated into the controllers themselves often after customizing the parameters for specific users [11, 12, 17].

Other approaches being explored include collecting movement-related muscle activation patterns in able-bodied subjects and then using machine learning methods to develop a more generalizable mapping between desired kinematics and the FES stimulation parameters needed to generate those motions [20]. Muscle activation patterns from able-bodied individuals can provide an informative starting point for controller development. However, those activation patterns may need to be adapted to specific FES users to account for alterations in a given user’s neuromusculature after injury and due to their hardware and surgical constraints typically limiting FES activation to only a subset of the normally available muscles.

Virtually all FES control strategies being explored incorporate some empirical characterization of the user’s limb to customize the controller to the given person’s unique limb properties and FES system. Since the movement resulting from a given stimulation pattern will differ depending on where the arm is in space, collecting this empirical data often requires placing the arm in different locations and measuring the forces or torques required to hold the arm there with and without stimulation to characterize active and passive forces/torques [11, 12, 14, 16]. Subject-specific arm dynamics have been estimated by measuring forces required to move the arm along predefined paths [13]. Motion tracking systems and sensored robots have been used in combination to move and measure the arm [12–14, 18]. Alternatively, an appropriately-sensored exoskeleton can collect both kinematic and torque/force data, and the motors can be used to help place the arm in the different parts of the workspace for initial empirical data collection [11, 16].

Many studies have incorporated feedback into the arm FES control strategy [7, 11, 12, 14, 16–18]. However, feedback control requires sensors on the limb to compare the arm’s actual configuration with its intended configuration. Since people with a spinal cord injury at the C1–C4 cervical level often have some denervation and weakness, especially in the shoulder muscles, combining FES with a mobile arm support or exoskeleton—in some cases—may be essential for restoring arm function while also providing a convenient way to incorporate sensors for feedback control.

While well-executed feedback control has the potential to generate good quality arm movements, oscillations and instability can occur if the controller is trying to make corrections faster than the FES activated arm can respond. Here increasing the limb’s stiffness and damping parameters has been shown to improve stability [17].

Another form of feedback control—visual feedback from the FES user—is also likely to play a key role in reducing movement errors with practice. In systems where the user provides a continuous movement command to the FES controller, *consistent* deviations between the actual and desired movements can result in the user learning to make feedforward compensatory adjustments in the command signal. Random *unpredictable* deviations could also be corrected using visual feedback by making online error corrections in real time. However, just as automated feedback control algorithms can run into stability issues due to response delays of the FES system [17], error correction by the user through visual feedback can run into stability issues under different delays and response characteristics of the system being controlled [24]. Any arm FES controller will need to be tested to make sure it is stable when the user sending it movement commands has visual feedback and naturally tries to adapt its command output to compensate for perceived movement errors.

### 1.2. Combining FES with a brain–machine interface (BMI)

FES systems for restoring hand function often make use of retained shoulder or wrist motions to control hand grasp [1, 5]. However, individuals with spinal cord injuries at the first through fourth cervical levels have fewer movements they can make under volitional control, which limits the available options for conveying to the FES controller how they want their arm and hand to move. In these individuals, BMI technology can provide the needed high-degree-of-freedom command signals by extracting one’s desired arm and hand movements directly from the brain. Combining an intracortical BMI with an arm and hand FES system has the potential to restore movement by thought to individuals with the most debilitating forms of paralysis.

Although there have been limited demonstrations to date of intracortical BMI control of upper limb FES in humans [8, 19, 25], these few examples illustrate a range of testing and control strategies. One group, focused on restoring hand and wrist function to a person with a spinal cord injury at the C5–C6 cervical level, used machine learning techniques to identify a workable set of stimulation patterns through a trial-and-error exploration of the stimulation space [25].

Intracortical BMI control of shoulder and elbow using a feedforward/feedback control strategy has been demonstrated using a real-time simulation of an arm by a person who had lost all volitional arm movement due to a brainstem stroke [8]. Because the virtual arm was entirely simulated, the control system had real-time information of the arm’s state for use in feedback control. However, to translate feedback control methods beyond simulation and into clinical practice, sensors will be needed to track the arm state. Therefore, feedback control methods may be best suited to FES systems combined with exoskeletons, where sensors in the exoskeleton can gather and use information about arm state in real time [11, 16].

Intracortical BMI control of the full arm and hand reanimated by FES has now moved beyond the simulated arm and has been demonstrated in a person with an injury at the fourth cervical level. In this study, a simple empirically defined ‘lookup-table-based’ FES controller was used [19]. Here, preliminary calibration data was first collected by applying different muscle stimulation combinations until options were found that placed the arm in different useful configuration across the workspace. The recorded stimulation values and resulting joint angles were organized into independent ‘lookup tables’ for different joints. To control the arm in real time, a new desired limb configuration was extracted from the BMI every 50 ms, and the stimulation values from the lookup table that should achieve the desired joint angles were applied. Note, this simple method did not account for complex interactions between degrees of freedom. Therefore, the arm movements were not perfect. However, the study participant was still able to use visual feedback and the simplified control scheme to feed himself, demonstrating the potential of even a simple upper limb FES controller to restore one’s ability to perform activities of daily living.

### 1.3. Managing limb stiffness in FES

While kinematic variables, such as desired velocity or position, are commonly decoded in BMI systems for cursor or robot control, limb ‘stiffness’ or ‘impedance’ is a critical factor when devising a brain-controlled FES system. Limb stiffness can be modulated by varying the degree of simultaneous activation of agonist–antagonist muscle groups (i.e. cocontraction) and is affected by the tension within the activated muscle and tendon. Able-bodied individuals naturally vary the amount of cocontraction used throughout reaching without consciously thinking about it. People also volitionally modulate limb stiffness when the situation calls for more limb stability, such as when holding a hot cup of coffee in a crowded elevator or when stabilizing an umbrella in the presence of unpredictable wind gusts. Animal studies have shown that variations in arm stiffness can be decoded from the cortex [26, 27], opening up the possibility of incorporating volitional stiffness modulation into brain-controlled upper-limb FES systems.

Optimizing the degree of limb stiffness is particularly important for FES users because excess muscle cocontraction can lead to more rapid drain of the stimulator batteries and rapid fatigue of muscles that are often out of condition from disuse. Too little cocontraction makes the arm less stable and more susceptible to perturbations, which may not be detected and appropriately corrected for in individuals who have lost sensory feedback [28].

Simulations of postural stability around the knee have demonstrated how stability can be a function of both intrinsic muscle properties (aka short range stiffness based on the tension and spring-like characteristics of the muscle and tendon under different degrees of activation and stretch) as well as the degree of cocontraction of flexor/extensor muscles [6]. Some upper limb FES simulation studies have also explored modulating cocontraction as well as the intrinsic short-range stiffness parameter in the muscle model to improve upper limb force control and stability in a model arm [9, 10]. The value of incorporating additional biceps and triceps cocontraction into one’s FES feedback control was demonstrated using able-bodied individuals with surface stimulation [17] to illustrate cocontraction’s role in resisting perturbations. That study showed that joint stiffness and damping increased linearly with increasing stimulation of the antagonist muscle pair.

While a wide range of studies now show the importance of managing limb stiffness when restoring arm function via FES, how stiffness modulation is integrated into the control scheme will depend on the type of controller used. Although there has been a lot of progress in developing more sophisticated and accurate FES controllers using models, simulations, and limited human testing, it is unclear how much the added complexity will be useful in BMI-controlled FES system once visual feedback and an adaptive plastic brain is put into the control loop. Additionally, for translating FES systems to routine clinical practice, developing simple strategies that are easy to implement by non-expert technicians may facilitate the translation of BMI-controlled FES to clinical practice across more institutions.

Prior simulation work has showed FES-controlled arm movements could be improved by *automatically* varying limb stiffness as a function of variables derived from the kinematic command signals already being decoded from the BMI in real time [28]. That study showed how overall muscle energy expenditure was reduced and movement accuracy improved simply by automatically decreasing cocontraction when the decoded speed is increasing and then increasing cocontraction when the decoded command is trying to slow the arm down. That proof-of-concept study optimized a formula for automatically modulating the degree of cocontraction during reaching using just the desired velocity information already being decoded from the BMI. Automatically adjusting limb stiffness based on desired kinematic information already being extracted from the BMI simplifies the process of incorporating automated stiffness modulation into BMI-controlled arm FES systems.

### 1.4. Study goals

Here we bridge the gap between the simple lookup table controller method demonstrated in the first intracortical BMI-controlled full arm and hand FES system [19] and the numerous more complex FES control options under development. We lay out a straightforward, clinically feasible process for calibrating an upper-limb FES control system that expands on the previous lookup-table methods by now optimizing stimulation across all joints and muscles simultaneously for better coordination of multi-joint movements. We also incorporate stiffness as an independent degree of freedom, which will enable future testing of volitional control of limb stiffness using stiffness signals decoded from the BMI, although here we demonstrate its use with automated stiffness parameters that would be derived from a BMI’s kinematic commands. Furthermore, we show how to optimize stimulation parameters for this controller both in the simple case when each electrode activates a single muscle or group of muscles as current is increased versus the more challenging case when current spread causes different muscles with different lines of action to become active as stimulation is increased. Finally, we show how to further modify the base calibration process to arbitrarily shift how work is distributed across muscles.

Our simplified method uses the common, energy-efficient cost function of minimizing the muscle forces squared. However, there are many reasons one may want to deviate from this cost function and shift how stimulation is distributed across muscles (e.g. if high stimulation in a given muscle is causing stimulation artifacts or pain, or if some muscles are fatiguing more quickly than others). Here we also provide methods for arbitrarily redistributing work across muscles and demonstrate how this process could be utilized to improve performance in the case where some muscles fatigue faster than others by redistributing some work from the easily fatigued muscles to the more fatigue-resistant muscles.

Together, these improvements to the lookup-table control method should create more accurate and stable BMI-controlled arm movements over previous lookup table iterations. Studies are still needed to determine if simple control schemes like this one are more easily learned and adapted to by a BMI user compared to more complex options where both the controller and the user have feedback and are both simultaneously trying to correct for movement errors. Here we demonstrate a testbed where these BMI learning factors can be further explored. Additionally, this straightforward process for implementing a BMI controlled-FES system is designed to be easily implemented in clinical practice to further translation of brain-controlled upper limb FES technologies to more institutions and user.

## 2. Materials and methods

### 2.1. Study overview

The FES control process refined here is an extension of the ‘lookup-table-based’ method used in the first demonstration of a BMI-controlled arm and hand FES system in an individual paralyzed due to a spinal cord injury at the fourth cervical level [19]. In that initial lookup table method, preliminary calibration data was collected by applying different muscle stimulation combinations and measuring the resulting joint angles. To use these lookup tables for FES control, at each 50 ms decoding loop, the muscle stimulation values needed to achieve the newly decoded desired arm joint angles and hand aperture would be ‘looked-up’ and applied in real time to move the arm to the new decoded location. Given the time-consuming nature of populating these lookup tables via manually adjusting stimulation parameters and seeing where the limb ends up, interpolation between calibration points was used to estimate a continuum of values spanning the workspace.

Here we lay out an analytical approach that allows for the incorporation of a stiffness degree of freedom. Not only should incorporating a stiffness degree of freedom improve limb movements and stability [6, 10, 17, 28], defining stiffness reduces the stimulation search space to *a simple problem that is easily solved analytically*. Unlike the previous lookup-table demonstration [19], our method mathematically solves for all muscle activations across all degrees of freedom simultaneously. Therefore, it also efficiently addresses the challenge of accounting for muscles that impact more than one joint and/or multiple angles within the same joint and the challenge of distributing stimulation across ‘redundant’ muscles (i.e. muscles with overlap in their biomechanical effects).

To demonstrate our methodology, we simulated the process of building the lookup tables using a computational model of a human paralyzed arm activated via FES. The Dynamic Arm Simulator [7, 8] is a well-validated and publicly available FES musculoskeletal model built from cadaveric morphological parameters [29] with Hill-type muscle models [30, 31]. Six arm muscles were included—anterior deltoid, posterior deltoid, brachialis (Br), triceps lateral head (TS), triceps long head (TL), and biceps (Bi) forming a two-joint, six-muscle FES control system. Movements were limited to two dimensions within the horizontal plane as if moving along a frictionless tabletop. Additional model details have been reported elsewhere [7, 8].

**Figure 1.**
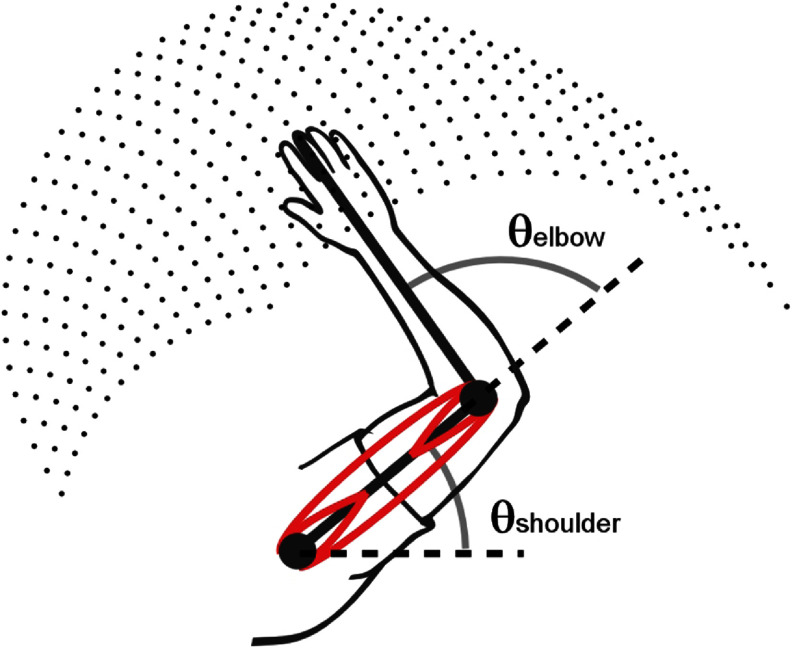
The two-joint, six-muscle example system used in this simulation study. This system contains redundant and biarticular muscles for both flexion and extension directions at both joints. Red lines indicate four monoarticular muscles (anterior and posterior deltoids, brachialis, triceps lateral head) and two biarticular muscles (biceps and triceps long head). The two joint angles allow for movement in the horizontal plane as if moving along a frictionless tabletop. Lookup tables were built for a workspace covering all combinations of shoulder and elbow angles ranging from 15°–85° and 20°–130° respectively. Dots show the workspace in 5° increments.

The model system is illustrated in figure 1. It is the simplest arm model that includes both ‘redundant’ muscles (i.e. muscles with overlapping biomechanical effects) and biarticular muscles (i.e. muscles that impact more than one joint simultaneously) to highlight our method’s ability to appropriately distribute stimulation across multiple muscles and coordinate activity across multiple joints. Although we limited movements to six muscles and two joints in the horizontal plane for this demonstration, the methods are designed to be expandable to include any number of muscles and joint angles as needed to represent the actual arm system being controlled.

As with virtually all FES control systems, some empirical data about the arm’s response to stimulation is needed to parameterize the control system for a given user. In this study, simulations were run using different amounts of initial empirical data to quantify how the amount of initial data used to build the lookup tables impacted final movement accuracy.

Calibrating a simple lookup-table-based FES controller can become challenging when additional muscles with different lines of action become activated with increasing current levels. This problem is often seen in nerve cuffs containing multiple small contacts intended to stimulate individual fascicles. Spillover of current to neighboring fascicles can lead to complicated current-to-muscle-force/torque recruitment curves [32, 33]. Current spillover can cause similar challenges with surface, epimysial, or intramuscular electrodes if neighboring muscles with different lines of action become activated as current is increased (see example in [20]). Here we illustrate an iterative process that can be used to account for these more complex recruitment curves while still maintaining a simplified matrix framework for simultaneously solving for all joint torques as a function of all stimulable muscles/electrode contact. Simulations were run for two different spillover scenarios to demonstrate this iterative process, and the number of iterations required to converge on a solution was quantified. Residual errors in the solution to the system of equations are also reported to further characterize how well the iterative process works.

Although we use a popular cost function for distributing work across muscles (i.e. minimizing the sum on the muscle activations squared), there may be times when a user may want to override this default distribution of work, such as if stimulation of certain muscles are more likely to cause pain or generate recording artifacts in the BMI, or when some muscles fatigue at a faster rate than others. Here we illustrate a process for arbitrarily modifying the distribution of work using the example where we want to shift the workload to rely more heavily on the fatigue-resistant muscles and less on the easily fatigued ones. Through simulations, we then demonstrated the effectiveness of this process by: (1) showing how both lookup tables generate the same accurate initial positions and (2) comparing how position accuracy declines over time as muscles become fatigued.

In total, the simulations reported here provide a roadmap to: (A) incorporating stiffness modulation into a simplified upper-limb FES controller, (B) distributing stimulation across redundant and biarticular muscles to minimize energy usage and coordinate activity across joints, (C) adapting the analytical methods for when current spillover activates additional muscles with different lines of actions at increasing current levels, and (D) modifying the distribution of work across muscles when desired using the example of improving position accuracy over time when some muscles fatigue more rapidly than others.

### 2.2. Theoretical approach

In prior human BMI studies, effective stimulation values for achieving different joint angles were found via manual trial and error [19] or a machine learning search process [25]. Those same resulting joint angles could have been achieved using an infinite number of possible combinations of stimulations representing different amounts of cocontraction of agonist–antagonist muscle groups—a parameter that effectively varies limb stiffness. Therefore, by defining our desired limb stiffness at each decoding loop, we significantly reduce the number of possible combinations of stimulation that could achieve the desired joint angles. Here we demonstrate an analytical method for determining the stimulation values that will put the arm in a given configuration with a specified level of stiffness defined here as the degree of opposing torques around a joint angle. Importantly, with this method, determining the stimulation values for a whole range of different stiffness levels is trivial once the equations are parameterized for a given limb configuration.

In a prior proof-of-concept study that developed guidelines for modulating stiffness levels throughout a movement using decoded kinematics from a BMI [28], stiffness metrics were approximated as the degree of cocontraction of agonist/antagonist muscle pairs using a simplified arm model with only one flexor and one extensor at each joint. However, that definition does not account for the impact of passive torques, and that definition becomes less clear when there are redundant muscle groups and not just a single pair of muscles. In this study we define our desired stiffness (*S*) in units of balanced opposing flexion/extension torques—a definition that can more appropriately accommodate passive torque contributions and any number of muscles acting on a given joint angle. To stably maintain any given desired joint angle, the net torque around that joint angle must be zero. This state is achieved by ensuring all passive plus active flexion torques (*Pf + Af*) exactly balance all passive plus active extension torques (*Pe + Ae*) as in equation (1)

\begin{equation*}S = Pf + Af = - 1*\left( {Pe + Ae} \right).\end{equation*}

Here, passive torques refer to torques resulting from the inherent elastic properties of the muscles and connective tissue, and active torques are those produced by the activation of the muscle’s actin and myosin contractile elements via stimulation.

To modulate stiffness, the stimulation applied to different muscles would be scaled up or down as needed to achieve the desired balance of total opposing active plus passive torques. Note that both the passive and active torques vary as a function of joint angles. Therefore, to achieve a desired limb configuration and stiffness, one would simply calculate the set of muscle stimulation parameters that make equation (1) true when parameterized for the desired limb configuration as well as stiffness level.

To calculate how much activation is needed from each muscle to achieve the desired limb configuration and stiffness, the above principle is used to formulate a system of equations where there is one flexion equation and one extension equation for each joint angle being controlled. The form of the pair of equations is the same for each joint angle in the system and is illustrated generically in the equation set 2 below

\begin{equation*}S - Pf = Af\end{equation*}

\begin{equation*} - S - Pe = Ae.\end{equation*}

Equations (2.1) and (2.2) come from rearranging equation (1) where *S* is the desired stiffness in units of opposing torques, *Pf* and *Pe* are the net passive flexion and passive extension torques respectively, and *Af* and *Ae* are the net active flexion and active extension torques around a given joint angle generated by stimulation. The signs follow the right-hand rule for torques.

Equation set 2 can be further expanded by replacing *Af* and *Ae* by a sum of the active torques produced by stimulating each of the individual flexor and extensor muscles as shown in equation set 3

\begin{equation*}S - Pf = \mathop \sum \limits_{i = 1}^{N{\text{flexors}}} {{ }}\left( {Cf\,^*_{i}Af\_max_i{\text{ }}} \right){\text{ }}\end{equation*}

\begin{equation*} - S - Pe = \mathop \sum \limits_{i = 1}^{N{\text{extensors}}} \left( {Ce_{i}^*Ae\_max_i{{ }}} \right).\end{equation*}

Here, *Af_max_i_* and *Ae_max_i_* are the max torques the *i*th flexor or extensor muscle can produce respectively, and *Cf_i_* and *Ce_i_* are their respective scale factors indicating the proportion of each muscle’s max torque generated with stimulation. As is common practice when scaling to the maximum producible muscle torques, these scale factors are limited to between 0 and 1. For each individual desired joint angle and desired stiffness level, our goal is to find a set of achievable scale factors that will make equation set 3 true.

While solving for the scale factors in equation set 3 can identify muscle stimulations that will produce the desired stiffness for each individual joint angle, we also need to account for muscles that impact more than one joint angle. Coordination across joints and joint angles can be achieved by solving a larger system of equations generated by combining equation sets for each joint angle being controlled. Here we represent that larger system of equations generically in matrix notation

\begin{equation*}{\mathbf{x}} = {\mathbf{A}}{{*}}{\mathbf{c}}.\end{equation*}

This equation form is similar to what many other studies have used to simultaneously identify activation levels across all muscle that will produce the desired combination of joint torques. However, in our case, the number of rows in the ‘**A**’ matrix and ‘**x**’ vector is two times the number of joint angles being controlled because each joint angle contributes a separate flexion and extension equation (as described by equation set 3) to the system. Each individually stimulable muscle is represented by a column in the **A** matrix, and the values in the **A** matrix reflect that muscle’s maximum flexion or extension torque exerted on each joint angle being controlled (*Af_max_i_*, *Ae_max_i_*). Note at least half of the terms in the **A** matrix will be zeros as a muscle will only exert either a flexion or extension torque on a joint but not both, and most muscles will only exert force on a subset of the joint angles in the system.

**Figure 2.**
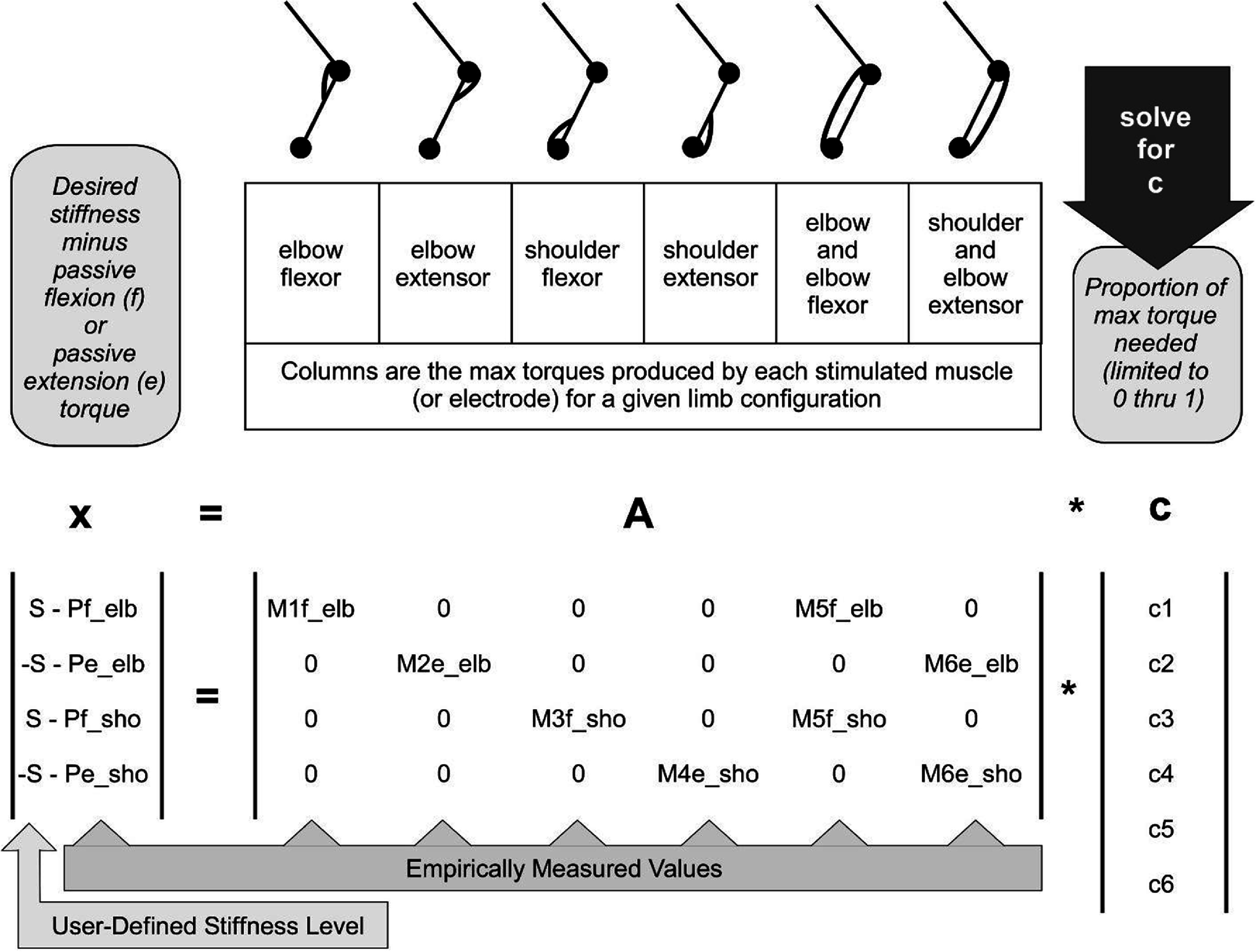
Components of equation (4) used in this two-joint, six-muscle proof-of-concept demonstration.

Figure 2 illustrates the system of equations used in this study for the two-joint, six-muscle system where two muscles span both joints. Solving these equations as a system ensures the resulting stimulation values account for the effects of the biarticular muscles on both joints simultaneously and accounts for the redundant muscles in the system. While the 2D arm model illustrated in figure 1 and the associated system of equations illustrated in figure 2 represent the simplified system used in this simulation study, it is important to keep in mind that the process and form of these equations can be expanded to any number of muscles and joint angles as needed.

Stimulating the muscles based on the scale factors in the **c** vector for this system of equations should put the limb into the joint angle and stiffness configuration that has the specified passive torques *Pe_i_* and *Pf_i_* for each joint angle and max active torques *Af_max*_*i*_ and *Ae_max*_*i*_ for each muscle. Therefore, the initial empirical data needed to generate this system of equations for different combinations of joint angles are the passive and active max torques measured at those joint angles (see [11–14, 16, 18] for how other studies have used robots and exoskeletons to collect empirical data for FES controller parameterization). Additionally, to generate lookup-tables in terms of stimulation parameters, such as pulse amplitude, additional empirical data is required on the relationship between the stimulation parameters used and the amount of active torque produces (see [11–13, 16–18] for alternative options for estimating these functions). An efficient process for collecting all needed empirical data and generating the final lookup table control system is described later in section 2.4.

### 2.3. Modifying the algorithm for current spillover

**Figure 3.**
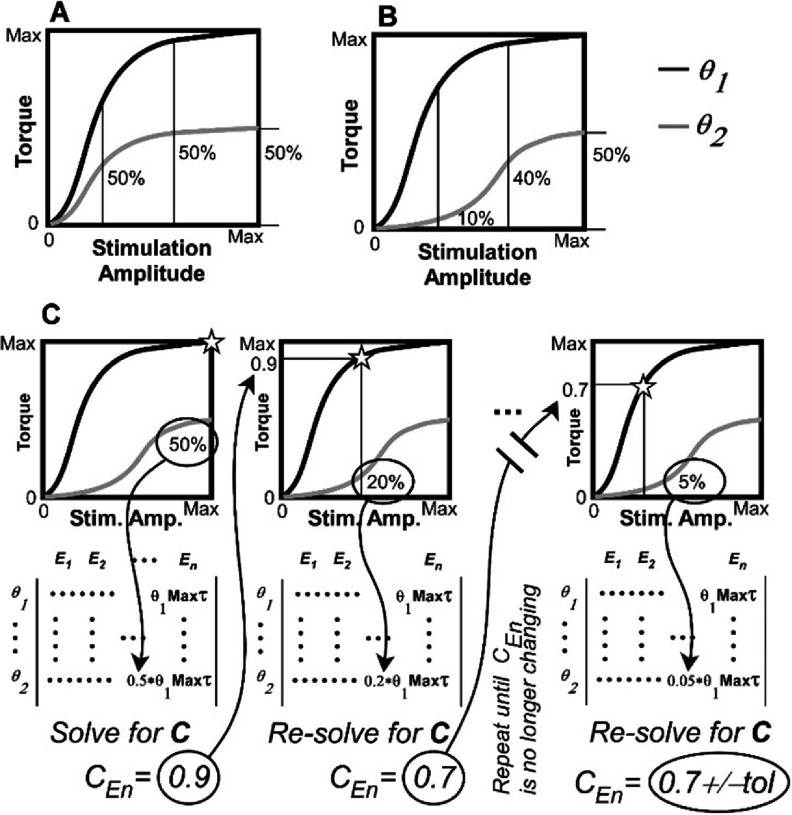
(A) An example stim.-to-torque relationship representing what is typically seen when a single activated muscle impacts more than one joint. Note how the active torque produced on joint angles *θ*_1_ and *θ*_2_ have a *consistent* ratio at all stimulation amplitudes for a given limb configuration. (B) This example illustrates stim.-to-torque profiles with *dissimilar* ratios for the two joint angles representing what often occurs when increasing stimulation through a single electrode contact starts activating additional muscles that add their own unique torque contributions to the profiles as current is increased. In this case, the ratio of active torques produced on joint angles *θ*_1_ and *θ*_2_ changes with the stimulation amplitude used. Part (C) illustrates an iterative process that can be used to solve our system of equations when the *ratio* of active torques on different joint angles varies as a function of stimulation level such as in (B). The star in each plot indicates the stimulation amplitude from the stim.-to-torque profiles at which the ratio of *θ*_2_/ *θ*_1_ is pulled and used in the **A** matrix (below each plot) for electrode ‘*n*’ (*E_n_*).

The system of equations derived in the previous section and illustrated in figure 2 assumes each muscle can be activated individually or, if it is a group of muscles activated by a given electrode, they at least act as a single consistent unit. In this scenario, the term ‘muscle’ or ‘electrode’ could be used interchangeably in the equations. However, for electrodes that are placed in or on peripheral nerves containing a mix of fascicles going to different muscles, stimulation through one electrode contact often starts activating additional muscles as the current is increased [32, 33]. Similarly, with some surface, intramuscular, or epimysial electrodes, current can sometimes spread and activate nearby muscles as current is increased. Figure 3 illustrates the challenge of this spillover effect in an example where two muscles are activated by one electrode contact. In figure 3(A), one electrode activates two muscles affecting different joint angles but with similarly shaped stimulation-to-torque profiles. Here, these two muscles can be treated as a single ‘muscle’ in our system of equations as long as all joint angles affected maintain a similar constant proportional relationship as stimulation changes.

Unfortunately, more often than not, when one electrode activates more than one muscle, the relationship between charge applied and the resulting joint torques will not be consistent across joints as in the example shown in figure 3(B). Here, as stimulation through the contact increases, additional muscles become activated each with their own unique stimulation-to-torque profiles. This difference in the stimulation-to-torque relationships will cause problems with the above system of equations if one were to simply use the max torque produced on each joint angle for a given electrode to parameterize the electrode’s column in the **A** matrix. Figure 3(C) both illustrates why this is a problem and how an iterative process can be used to solve the problem of identifying a single stimulation value for an electrode when current spread causes dissimilar activation profiles for different joint angles.

This iterative process starts like the process described in the previous section but with columns of the **A** matrix representing specific electrodes instead of specific muscles (*E*_1_, *E*_2_, *… E_n_*). At the start of the process, the **A** matrix is parameterized using the max torques that can be produced on each joint angle by each stimulating electrode just as before. In the example in figure 3(C), note that the max torque for *θ*_2_ is 50% of the max torque for *θ*_1_ on electrode *E_n_*. When solving for the **c** vector, we see that the initial solution to the system of equations wants us to scale the torques on *both* joint angles to 90% of their original max values. However, as the second plot in part C shows, applying the stimulation that produces 90% of the max torque in *θ*_1_ would produce an inadequate amount of torque in *θ*_2_ because *θ*_2_’s torque-producing ability is now only 20% instead of 50% of *θ*_1_’s torque producing ability at that lower level of stimulation.

To account for this change in the relative torque-producing ability, one would iteratively re-solve for **c** after replacing *θ*_2_’s max torque in the **A** matrix with its percent of *θ*_1_’s max torque at the stimulation level suggested by the previously calculated **c** which should be more representative of their relative ratios at the actual stimulation level to be applied. This process would be repeated until it converges on a consistent **c** value (plus or minus some small tolerance), which happens when the stimulation level defined by the solution to the system of equations produces the same relative torque ratios as was used to define the **A** matrix.

### 2.4. Practical implementation

The above system of equations requires collecting a fundamentally different type of preliminary data than what was used in the previous BMI-controlled human study. Instead of applying stimulation and seeing where the arm goes in a trial-and-error search process [19], this method uses measured joint-angle-dependent passive (*Pe_i_*, *Pf_i_*) and maximum active torques (*Af_max_i_, Ae_max_i_*) to parameterize **x** and **A** in equation (4) thus allowing us to *solve* for the stimulation parameter vector, **c**, at key points throughout the workspace. This process is more in line with the empirical data collection process used to calibrate some feedback controllers and could make use of similar equipment and processes [11–13, 16]. Once position dependent passive and max active torques are empirically determined for parameterizing **x** and **A**, the **c** vector can be calculated for a whole range of different stiffness levels, *S*.

Collection of these passive and active torque measurements can be performed using a sensored exoskeleton device, such as the commercially available Harmony SHR® (Harmonic Bionics, Austin, TX) or systems like those in [11–13, 16]. The sensored exoskeleton would collect the net torques required to hold the relaxed arm stationary in various sample limb configurations to quantify the joint-angle-dependent passive torques around each joint angle being controlled. If gravity contributes torque to any included joint angle, its effect would automatically be incorporated into that angle’s passive torque measurements. This collected passive torque data is used to parameterize the **x** vector.

To parameterize the **A** matrix, while still keeping the arm stationary via the exoskeleton, one would also measure the *additional* torque produced by each muscle individually as the amount of charge is incrementally increased until one has identified each muscle’s maximum active torque for a given set of joint angles. This step also serves to define the non-linear relationship between the stimulation parameter being varied (e.g. pulse amplitude and/or width) and the resulting active torques (like the examples in figures 3(A) and (B). In this study, we use complete torque profiles measured from the model arm across the activation range. However, methods for modelling these stimulation-to-muscle-torque profiles with sigmoid functions are available [16, 18].

This stimulation-to-muscle-torque relationship is used to iteratively solve for **c** in the case where one electrode activates multiple muscles nonuniformly as depicted in figure 3(C). In all cases, this relationship is also used later to define our lookup tables in terms of the actual stimulation parameters and not just scaled active muscle torques defined by the solution to our system of equations.

In our 2D six-muscle model arm illustrated in figure 1, the under-determined system of equations illustrated in figure 2 has multiple ‘correct’ solutions because there are redundant muscles for the different joints. We solve the under-determined system of equations using MATLAB’s *lsqminnorm()* function which finds the least squares minimum norm solution to a linear system of equations. This option finds the most accurate solution (i.e. least squares) while efficiently distributing work across redundant muscles by minimizing the ‘norm’ or Euclidean magnitude of the vector of coefficients in **c**. Distributing work across redundant muscles in this manner is a commonly used technique and is thought to minimize fatigue [34].

Once an initial least squares minimum norm solution for **c** is found, we then apply the additional constraint where all values are limited to between zero and one. If any scale factor in **c** is negative, that would suggest the ideal solution wants the muscle to produce an expansion force instead of a contractile force, which no muscle can do. Therefore, that muscle’s stimulation should be set to zero and one would simply recalculate the solution to the system of equations with that muscle removed from the **A** matrix. Similarly, if a scale factor in the **c** vector is above one, meaning the ideal solution requires a given muscle to produce more torque than it is capable of in that configuration, one could limit its scale to one simply by removing its column from the **A** matrix and subtracting those max torque values from the **x** vector before recalculating the least squares minimum norm solution. These steps should be repeated until no muscle scale factor in **c** is outside of the 0–1 range.

Once the ‘**c**’ vector is solved for, the previously determined torque-to-stimulation-parameter relationships are used to find the actual stimulation parameter (e.g. pulse amplitude and/or width) that will produce the desired torque defined by *c_i_* times the max active torque produced by muscle *i* in that limb position. Those stimulation values are used to populate an initial lookup table matrix **L_o_** of dimension *n*+ 1 × *m*. Here *m* is the number of muscles (or electrodes) being stimulated and *n* is the number of joint angles being controlled. The extra ‘+ 1’ accounts for the extra stiffness degree of freedom being controlled. Note, this setup assumes one always wants to increase/decrease the stiffness in all joint angles similarly to keep all parts of the arm at the same stiffness level. If desired, different stiffness levels could be chosen for different joints by adding dimensions to the lookup table and modifying equation (4) to accommodate different *S* values for different joints or joint angles.

The final step in the table-building process is to apply the theoretically optimal stimulation values and measure the actual resulting joint angles. This step is needed because there will inevitably be some amount of error in the measurements used to parameterize the **x** and **A** matrices (equation (4)) and in the measured torque-to-stimulation-parameter function. A sensored exoskeleton could again be used for this step by having it allow the arm to move freely and report out the actual resulting joint angles for each applied set of stimulation parameters. The final lookup table, **L**, is then populated by using the actual measured joint angles as a function of the applied stimulation and interpolating to fill in gaps between measured points. This lookup table could be refined further over additional testing sessions by repeating the above process for additional limb configurations and replacing interpolated data points with actual data points.

Table 1 summarizes all the steps needed to build the final lookup tables.

**Table 1.** Step-by-step process for building the lookup table that allows one to ‘look up’ the stimulation values to apply to each muscle (or electrode) to achieve a desired set of joint angles and limb stiffness.

1) Using a sensored exoskeleton, such as the Harmony SHR®, hold the arm stationary at different locations throughout the workspace and collect the following data: (a) Collect **passive torques** on each joint angle by recording the torques needed for the exoskeleton to hold the arm stationary in the current position. (b) Apply increasing levels of stimulation to each muscle individually and record the torques needed to keep the arm stationary in the current position as the stimulation increases. Continue to increase stimulation until the resulting measured torque stops changing or the stimulation is at its maximum safe level—whichever comes first. Subtract the previously calculated passive torques (1a) from this sequence of values to get the **stimulation-to-active-torque** relationship for each muscle in each location. (c) Record the **maximum active torque** in each joint angle for each muscle by taking the max torques from the stimulation-to-active-torque relationship calculated in 1b.

2) For each arm configuration *and* each desired stiffness level: (a) **Build a system of equations** using the passive torques from (1a) to populate the **x** vector and maximum active torque values from (1c) to populate the **A** matrix. Repeat the calculation using different stiffness values, *S*, in the **x** vector. (b) **Solve each system of equations** using *lsqminnorm()* to get the least squares minimum norm solution while limiting the scaling coefficients in the **c** vector to between 0 and 1.

3) **Generate an initial lookup table,** **Lo**, in terms of stimulation parameters by taking the calculated scaling coefficients **c** for each desired arm configuration and stiffness and using the stimulation-to-active-torque relationship from (1b) to define the stimulation parameters that will give the proportion of the max active torque defined by **c** needed to achieve each desired limb configuration and stiffness.

4) **Test the initial lookup table** by applying the recommended stimulation values and recording where the arm *actually* goes.

5) **Build the final lookup table, L,** using the actual arm positions collected in (4) and interpolation to fill in intermediate values as needed. Continue to refine the final lookup table by replacing interpolated points with actual measured points as more data is collected over time.

### 2.5. Modifying the lookup table to address problems such as fatigue

The least squares minimum norm solution to our system of equations inherently minimizes fatigue by distributing the work across redundant muscles using the minimum norm as a cost function [34]. However, under some conditions, one might want to manually shift how stimulation is distributed across redundant muscles, such as in the case where one muscle in a redundant pair is much more easily fatigued than its counterpart, or if stimulation of certain muscles produce more pain or recording artifacts than some other muscles available to perform the needed action. Here we show how the distribution of work across these redundant muscles can be intentionally manipulated simply by scaling down the max torque in the **A** matrix of any muscle you want to de-emphasize before solving the system of equations. The minimum norm solution will then put less work on the less desirable muscles and shift more work to the remaining more desirable muscles. Note, however, for the resulting lookup tables to still produce the correct limb configuration, this scaling must be accounted for when extracting actual stimulation values from the empirically derived stimulation-to-torque functions when generating the lookup tables. For example, if a given muscle had its max torque reduced by half in the **A** matrix, then the resulting scaling coefficient tells us to use stimulation parameters that will generate half the max torque times that scale factor. Equivalently, the scaling coefficient could simply be cut in half and applied to the full max torque value.

Here we illustrate the process of arbitrarily rebalancing the work across muscles using an example situation where one or two of our six muscles become fatigued and lose force producing capacity over time. While each muscle’s rate of fatigue during real-world use will depend on its complex temporal patterns of activation and rest specific to the tasks being performed, a good rule of thumb could be to base the shifted max torque values on anticipated (or measured) max torques seen during actual use after a typical amount of fatigue has set in. However, these fatigued max torque values should form the *lower bound* of adjustments to the **A** matrix. One may want to choose values in between the initial and fatigued max torque values to generate lookup tables that are a compromise between initial efficiency and fatigue resistance.

### 2.6. Simulations/tests performed

#### 2.6.1. Effects of sampling resolution

The two-joint, six-muscle computational arm model pictured in figure 1 was used to illustrate the above-described process for generating a joint-angle-to-muscle-stimulation lookup table for FES control. The lookup table was built for a workspace spanning shoulder angles of 15°–85°, elbow angles of 20°–130°, and stiffness levels spanning 3–13 Nm of opposing joint torques. Because simulations allow one to efficiently test many more arm configurations than is practical clinically, we were able to simulate the process of building a very detailed lookup table with (simulated) empirical data collected in 1° increments—an impractical resolution to collect clinically. To quantify the effect of lookup table sampling resolution, this high-resolution data was then down sampled by different amounts, and interpolation was used between sampled points to populate the lookup table in 1° increments. The accuracy of the limb placement and stiffness as a function of the amount of sample points used to build the lookup table was then quantified as the mean difference in the fingertip position and average shoulder and elbow stiffness between the full resolution and the interpolated down-sampled versions of the lookup tables. Note these ‘sampled’ position values reflect the final stable static position of the arm at the associated stimulation values which is independent of the starting position.

To illustrate the practical effects of the lookup table resolution, example trajectories were simulated using the lookup tables generated in 1°, 10°, and 20° increments. Additionally, example brain-controlled trajectories are also included from an able-bodied Rhesus macaque controlling the simulated arm using these same three lookup tables. The starting position for all these arm movements is illustrated in figure 1 with targets spaced radially 14 cm away. Average time to target was 1.3 s for both simulated and monkey-controlled trajectories. This lookup table control method requires updating the desired position and stiffness level at each 50 ms time loop. For the simulated movements by a simulated user, positions were chosen to mimic a typical bell-shaped velocity profile. For the simulated arm trajectories commanded by the non-human primate, 2D velocity was decoded from ∼52 cortical neurons in real time via a simple linear transfer function, and the decoded velocity was integrated over each 50 ms timestep to get the next desired position. For both the simulated and animal user, an appropriate stiffness level was determined at each timestep from the associated velocity based on previously reported methods [28]. All animal experiments were approved by the Cleveland Clinic’s institutional Animal Care and Use Committee.

#### 2.6.2. Iteration time require to account for current spillover effects

**Figure 4.**
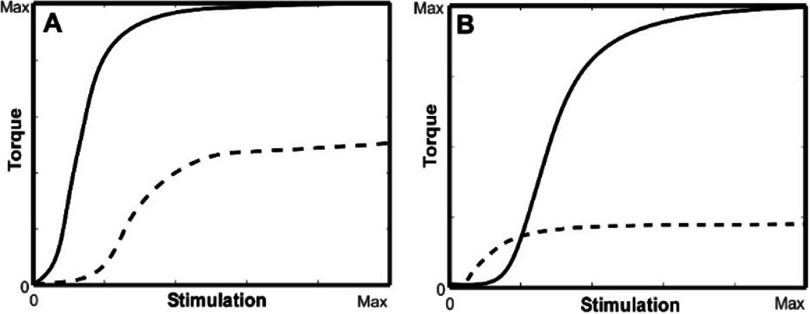
Two hypothetical examples where a single electrode activates two different muscles that impact two different joint angles in a non-uniform manner. Solid and dotted line reflect the elbow and shoulder flexion torques (respectively) produced as a function of stimulation level on the single electrode.

To validate that the iteration process depicted in figure 3(C) can solve the system of equations under the condition of current spread when a single electrode activates two muscles that non-uniformly affect two different joint angles, we generated and tested the two different stimulation scenarios. We replaced the torque on the shoulder and elbow from the biceps in our calculations with new torque profiles from two hypothetical monoarticular muscles (one effecting shoulder flexion and one effecting elbow flexion) using the non-uniform torque profiles illustrated in figures 4(A) and (B). These torque profiles reflect more common (4A) and less common (4B) situations that one might see when increasing current levels start to activate more than one muscle with different lines of action.

Iterative solutions were calculated for stiffness levels 3°–13°Nm and shoulder angles of 15°–85° and elbow angles of 20°–130° in 5° increments using the hypothetical shoulder and elbow torque profiles from figures 4(A) and (B) in each limb configuration. This process resulted in a total of 3795 separate systems of equations on which to test the iterative process for each of the two scenarios. A tolerance of ±0.001 was used as the stopping criteria which means the process was stopped when the difference between the *c_i_* value from the previous iteration (and used to determine the proportional relationship between the two joints in the system of equations) and the *c_i_* value in the current solution had a difference of less than ±0.001. The number of iterations needed to meet this criterion and the starting and final error magnitudes of the solution to the system of equations were recorded. Error at each iteration was measured as the norm of the difference between the flexion/extension torques needed on each joint (defined in the **x** vector) and the actual torques that would be produced if applying the stimulation values calculated at each iteration.

#### 2.6.3. Effectiveness of accounting for fatigue

To demonstrate how the lookup tables controllers can be arbitrarily rebalanced using the example of reducing the reliance on muscles that are easily fatigued, 11 different scenarios were simulated with one or two of the six muscles fatiguing at a high rate (table 2). Specifically, the fatigable muscles were simulated to lose 0.5% of their torque producing ability per second. Standard and fatigue-resistant lookup tables were generated under the assumption that the user would need to use their arm for a duration of ∼100 s before resting. Therefore, to build more fatigue-resistant lookup tables, the terms in the **A** matrix for the fatiguing muscles were scaled by 0.5 to reflect the anticipated loss of torque producing ability after 100 s. For each of the 11 different conditions, standard and fatigue-resistant lookup tables were generated with stiffness levels ranging from 3 to 13 Nm, elbow angles ranging from 20° to 130°, and shoulder angles ranging from 15° to 85° in 5° increments. Simulations were then run where we attempted to hold the arm stationary at each of these arm angle and stiffness combinations using stimulation values from the standard versus the fatigue-resistant lookup tables. The error in endpoint position and stiffness as a function of time were tracked and compared between the two lookup tables. At each time step, a Wilcoxon rank sum test was used to determine if the endpoint position and stiffness errors were significantly different between the two lookup tables.

**Table 2.** Adjustment factors for 11 simulated example scenarios in which either one or two of the six muscles had a high fatigue rate. Terms in the **A** matrix for each muscle were adjusted before solving the system of equations by multiplying that muscle’s max active torque values by 0.5 as indicated below. Dashes indicate muscles that did not fatigue in each scenario. The 0.5 value represents the anticipated reduction in active-torque-producing ability in the fatiguing muscles after100 s of use.

*Example scenario #*	*1*	*2*	*3*	*4*	*5*	*6*	*7*	*8*	*9*	*10*	*11*
Shoulder flexor (Anterior deltoid)	0.5	—	—	—	—	—	0.5	—	0.5	—	—
Shoulder extensor (Posterior deltoid)	—	0.5	—	—	—	—	—	0.5	—	0.5	—
Elbow flexor (Brachialis)	—	—	0.5	—	—	—	—	0.5	0.5	—	—
Elbow extensor (Triceps lateral head)	—	—	—	0.5	—	—	0.5	—	—	0.5	—
Sho + Elb flexor (Biceps)	—	—	—	—	0.5	—	—	—	—	—	0.5
Sho + Elb extensor (Triceps long head)	—	—	—	—	—	0.5	—	—	—	—	0.5

## 3. Results

### 3.1. Accuracy as a function of sampling resolution

Collecting empirical data for calibrating an FES control system can be time consuming regardless of the control method used. Table 3 illustrates why collecting empirical data at a high resolution is impractical and why sparse sampling plus interpolation is a more practical and efficient option to use clinically. Specifically, table 3 shows the total number of arm positions at which passive and active torque data would need to be collected if sampled in 1°, 5°, 10°, 15°, and 20° increments throughout our defined 2D workspace.

**Table 3.** Total number of data points at which empirical data would be collected as a function of angular sampling resolution for our two-angle system with 15°–85° of shoulder rotation and 20°–130° of elbow rotation.

Angular sampling resolution				
1°	5°	10°	15°	20°
7881	345	96	35	24

**Figure 5.**
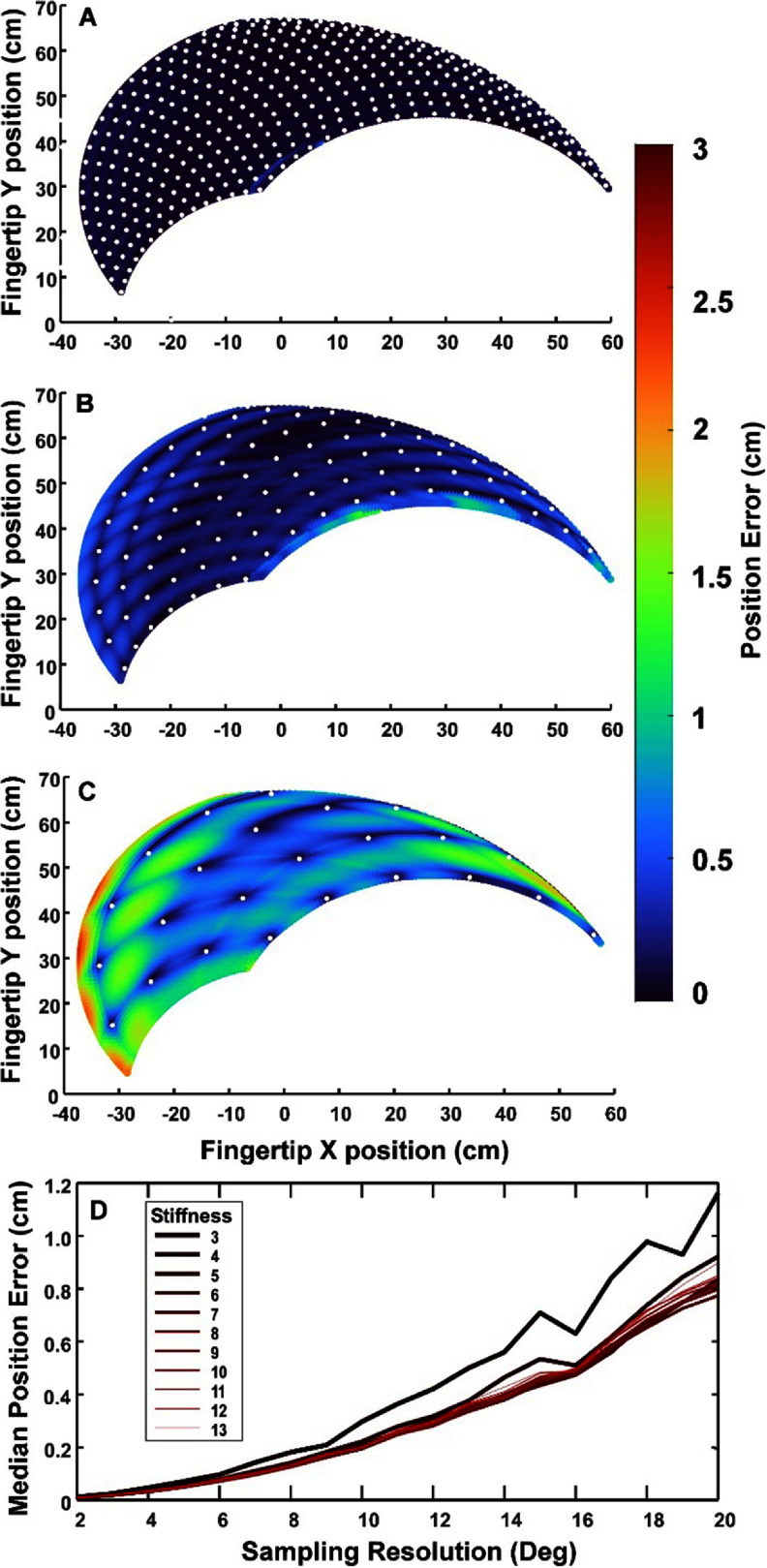
Fingertip position error magnitude (cm) when using lookup tables generated with different amounts of empirical data. White dots indicate fingertip positions for empirical data points collected at (A) 5°, (B) 10° and (C) 20° elbow and shoulder increments. Sampling locations have an error magnitude of zero by definition. The color at each in-between location shows fingertip positional error magnitude for each intended fingertip location across the workspace. These errors result from interpolating between empirical data points to estimate the needed stimulation values (examples shown are for a stiffness level of 8 Nm of opposing flexion/extension torque). (D) Median fingertip position error (cm) as a function of empirical sampling resolution at stiffness levels 3–13 Nm.

Figure 5 illustrates the effects of collecting empirical data on lookup-table accuracy at a range of different angular sampling increments. Errors are incurred when interpolating the needed stimulation values between points calculated using empirical data. Errors are reported here in terms of how far the endpoint (i.e. tip of the index finger) was from where it should have been when using the lookup table’s stimulation values. Note the largest errors are at the extreme edges of the workspace where passive torques vary more rapidly as a function of joint angle and, in some cases, extrapolation was needed.

Data in figures 5 (A)–(C) reflect errors at a stiffness level of 8 Nm. Figure 5(D) shows the median fingertip position error as a function of sampling resolution for all stiffness levels ranging from 3 to 13 Nm.

**Figure 6.**
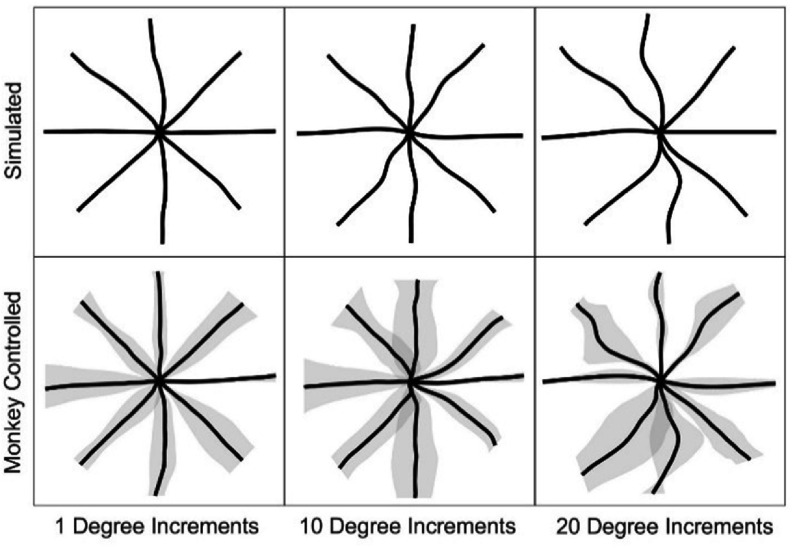
Example center-out fingertip trajectories of the simulated arm generated using the lookup tables calibrated in 1°, 10°, and 20° angular increments. The top row shows trajectories commanded by a simulated user. The bottom row shows mean (black) and two standard deviations (grey) of the trajectories generated by a Rhesus macaque using its intracortical signals to control the simulated arm in real time.

To illustrate the functional impact of the lookup table resolution, example fingertip trajectories of the model arm are shown in figure 6. Trajectories in columns one through three were generated using lookup tables built in 1°, 10°, and 20° increments respectively. The top row shows center-out trajectories generated by a simulated BMI user with a perfectly decoded velocity command generated using previously reported methods [28]. In this simulated BMI user, real-time correction of trajectory errors via visual feedback was simulated by reorienting the intended velocity vector toward the target at each time loop prior to integrating over each timestep to get the next desired position as in [28].

The bottom row shows mean (black) and two standard deviations (grey) of the closed-loop trajectories from a Rhesus macaque on the first day of learning to control the model arm via the lookup tables at the three different resolutions. Intracortical firing rates of ∼52 neural units were decoded into a desired 2D cursor velocity in real time via a simple linear transfer function. The desired velocity command was integrated at each timestep to get a desired position and converted to joint angles before using the different lookup tables to determine how much stimulation to apply to the arm model in real time. The arm model’s fingertip position was displayed back to the animal in the form of a moving cursor on a screen. Although these trajectories naturally contain more variability than the fully simulated ones in the top row due to decoding imperfections, they are included here to illustrate use of the lookup table controller in a model system with actual visual feedback—an important factor for this simplified FES control method which does not contain any embedded feedback in the controller itself.

### 3.2. Iteratively solving for current spillover effects

**Figure 7.**
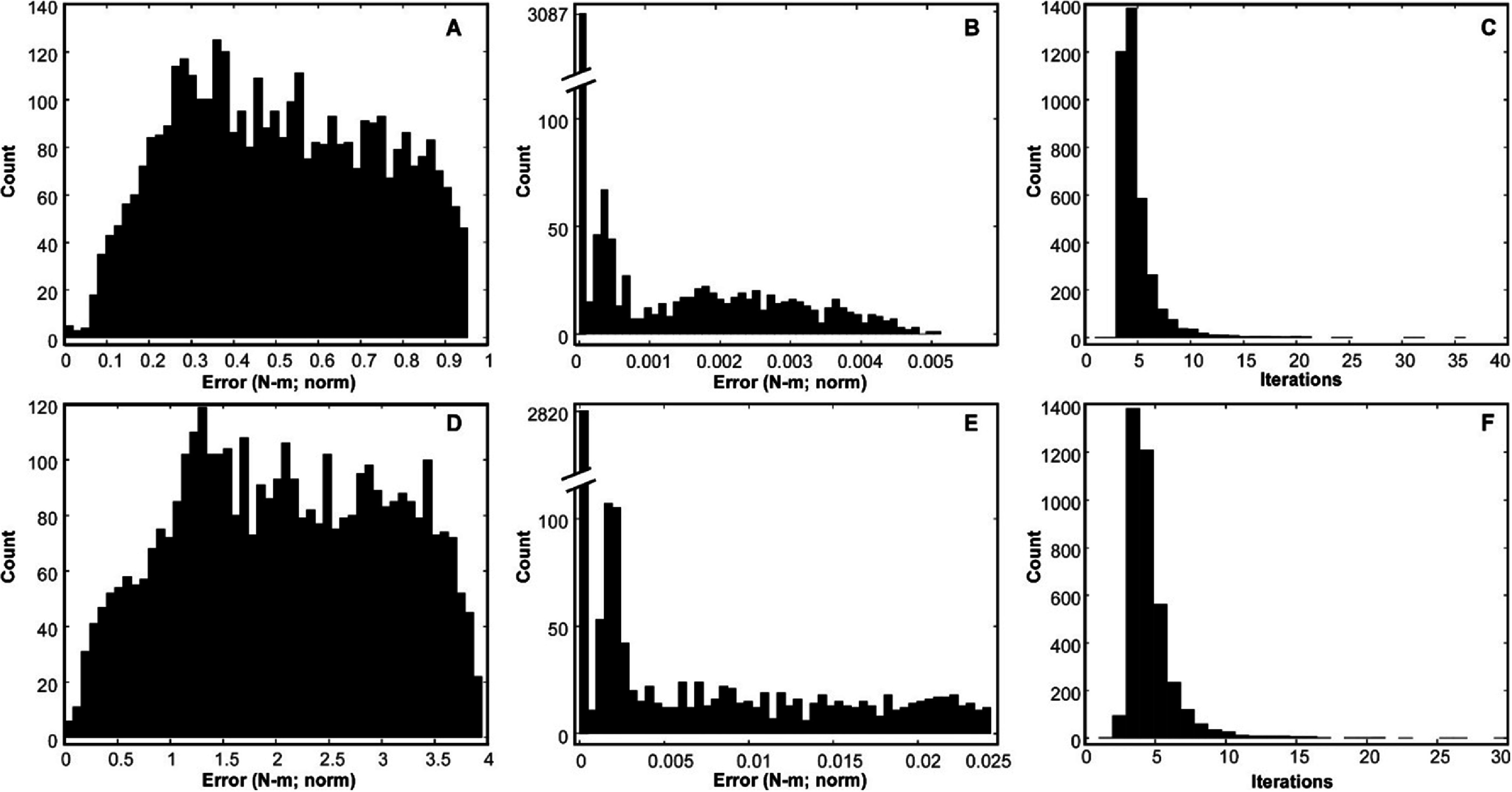
Parts (A) & (D) show the initial errors in active torque produced if applying our standard calculation methods to the scenarios depicted in figures 4(A) and (B) (respectively). Parts (B) and (E) show the errors in active torque produced after using the iterative process to solve the system of equations more accurately. Numerical values reflect the norm of the four errors in torque calculated for both flexion and extension of the shoulder and elbow. Parts (C) and (F) show the number of iterations required to converge on a stable **c** vector with a difference of less than ±0.001 Nm (norm) between iterations. Data shown include all combinations of stiffness levels 3–13 Nm, and elbow angle of 20°–130° and shoulder angle of 15°–85° in 5° increments (3795 total combinations). Out of the 3795 different system of equations iteratively solved, only four from the scenario depicted in figure 4(A) and one from the scenario depicted in figure 4(B) failed to converge and was stopped after 1000 iterations (not included in the histograms).

Figure 7 shows how well our iterative process worked to identify a set of electrode scaling coefficients that would produce the desired balance of active torques when a single electrode activated two muscles affecting two different joint angles with the hypothetical non-uniform relative activation profiles depicted in figures 4(A) and (B). Results shown are from 3795 separate systems of equations covering stiffness levels 3–13 Nm, and elbow angles 20°–130° and shoulder angles 15°–85° in 5° increments. Parts A & D of figure 7 show how the standard system of equations (which was our starting point in the iteration process) produced a considerable amount of error particularly in the complex scenario illustrated in figure 4(B). However, the iteration process converged on a successful set of stimulations with negligible errors a vast majority of the time (note the break in the scale bars in figures 7(B) and (E) and the small *x* axis scale). The last column of figure 7 shows the number of iterations needed to solve the system of equations to within the ±0.001 tolerance. Most of the configurations converged within five to ten iterations which posed no computational barrier. Only four combinations for the scenario depicted in figure 4 (A) and one combination for the scenario for figure 4(B) failed to converge and were terminated at 1000 iteration (not included in the histograms). Even still, their final error magnitudes were not large (i.e. 0.004–0.119 for scenario 4A; 0.216 for scenario 4B), and they only occurred in limb configurations with high stiffness levels and at the extremes of the joint ranges where the muscles can already have difficulty producing the high torque levels needed to maintain the desired position and stiffness.

### 3.3. Benefits of fatigue resistant lookup tables

**Figure 8.**
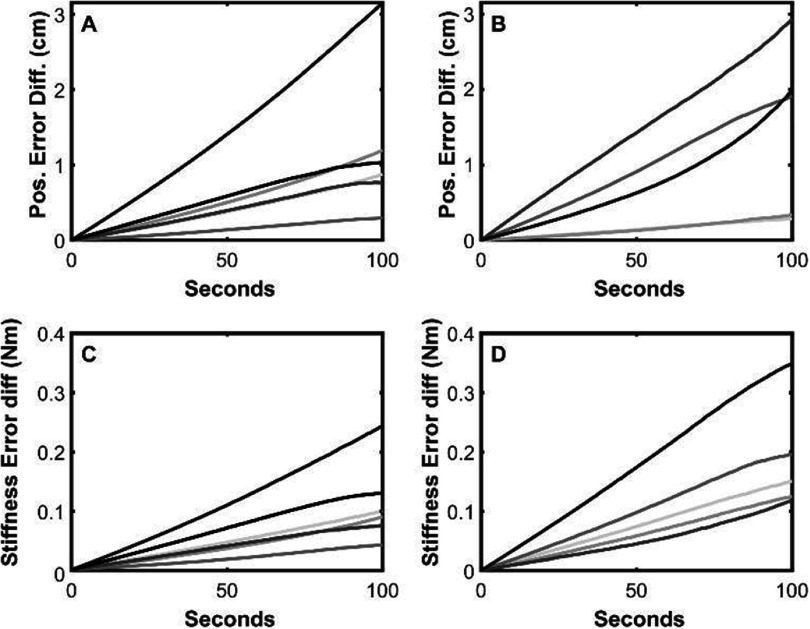
Comparison of fingertip position errors (A), (B) and stiffness errors (C), (D) produced over time when using standard versus fatigue-resistant lookup tables. Lines indicate the *difference* in median errors between methods (i.e. standard minus fatigue-resistant lookup table results). Median error lines were calculated across all combinations of stiffness levels and joint angles (3795 combinations per condition). The individual lines reflect the 11 different fatigue scenarios listed in table 2. The six lines in A & C are for the scenarios with one easily fatigued muscle (scenarios 1–6; darkest to lightest respectively) and the five lines in B & D are for the scenarios with two easily fatigued muscles (scenarios 7–11; darkest to lightest respectively). Note, at all timepoints except *t* = 0, all differences are significantly positive (*p* < 0.05 based on Wilcoxon ranks sum test) indicating the fatigue-resistant lookup tables consistently produced significantly lower errors than the standard lookup table.

Figure 8 illustrates the *difference* in the magnitude of the fingertip position error (A and B) and the stiffness error (C and D) over time when using the standard lookup table versus the ones designed to be more fatigue resistant. For easier viewing of the lines, results from simulations with one versus two rapidly fatiguing muscles are plotted separately on the left (A and C) and right (B and D) respectively. Note, at time zero, there are no errors in either type of lookup table because all tables were generated from equally valid solutions to our original system of equations. The benefit of using the fatigue-resistant lookup tables only becomes apparent over time when fatigue progressively reduces the torque producing ability of the more-easily fatigued muscle(s). At all timepoints beyond time zero, all scenarios had larger median (shown) and mean (not shown) position and stiffness errors when using the standard lookup table versus the fatigue-resistant lookup tables.

The standard lookup table process produces the most energy efficient stimulation parameters based on our original cost function (i.e. scaling coefficient vector **c** is the minimum norm solution to the system of equations). However, when building more fatigue-resistant lookup tables, the scaling process used to skew the system of equations produces a set of scaling coefficients that is no longer the minimum norm solution to the original system of equations. Averaging across all scenarios tested, the norm of the fatigue-resistant scaling coefficients (adjusted **c** vector) was 0.0411 larger (±0.0367 standard deviation) than the norm of the original **c** vector. This increase reflects how our adjustment process shifted more of the work to the non-fatiguing muscles and less work to the easily-fatigued muscles.

## 4. Discussion

FES has the potential to restore use of the upper limb to individuals with paralysis associated with high-level cervical spinal cord injuries. However, each paralyzed arm is unique with differing amounts of denervation and muscle atrophy. Therefore, the type and placement of stimulating electrodes need to be customized to the individual user. Once implanted, developing a custom control system for each user’s unique arm and FES system can be a daunting task. In this paper, we laid out a simplified analytical approach to generating an upper limb FES control system that could be applied to almost any user and FES system using an automatable calibration process for easy clinical implementation. We then illustrated this approach through a series of simulations spanning both systems with independently stimulable muscles and the more complicated situation where an electrode can start activating additional muscles with different lines of action as current is increased.

In upper-limb FES, any given arm configuration can typically be achieved with a nearly infinite number of muscle stimulation combinations. The large number of possibilities stems from the fact that the degree of cocontraction of antagonist muscles can be varied and the distribution of work across redundant muscles can be varied. Our approach manages this large number of possible solutions first by making limb stiffness, which is a function of cocontraction, a defined degree of freedom to be controlled. Second, we make use of the common and energy efficient ‘minimum norm’ cost function to distribute stimulation across redundant muscles, as this cost function is thought to reduce muscle fatigue and promote endurance in redundant muscle systems [34]. Our methodology takes all joint angles into consideration at once, which addresses the additional challenge of coordinating the effects of stimulation across multiple joints. This multi-joint coordination was demonstrated by the 2D arm model, which included biarticular muscles that span both shoulder and elbow joints. Although we limited the arm to a simple six-muscle two-joint-angle system for this demonstration, the process described here could easily be expanded to include additional joints and joint angles simply by adding two additional opposing flexion/extension torque equations for each additional joint angle to the system of equations.

Volitional stiffening of the arm has been decoded from the motor cortex of macaques [26, 27] suggesting that it may be possible to decode a volitional stiffness command from humans using an intracortical BCI system. While volitional control of stiffness would certainly be useful, dedicating cognitive and computational resources to extracting a volitional stiffness command from the brain is *not required* for one to take advantage of our FES control method. Previously published studies showed how an appropriate stiffness degree of freedom can be *inferred* from the decoded end-point velocity command, and how doing so can make movements more energy efficient and/or more accurate [28]. Specifically, that study showed that energy expenditure and accuracy of performance was optimized by reducing stiffness when the user was trying to accelerate (typically at the start of a reach) and then increasing stiffness when the user was trying to decelerate the limb (typically as the limb slowed down to approach its target). By making stiffness an independently modulable degree of freedom in the lookup table, beneficial stiffness can be *automatically* modulated as in [28], and—if decoded—additional *volitional* limb stiffness can be included on top of the automatically determined stiffness level. This would give the user the ability to stiffen the limb when desired but not require the user put in cognitive effort to optimize stiffness during regular arm movements.

Calibrating any type of FES controller requires collecting empirical data on the limb’s response to stimulation. Table 3 illustrates how rapidly the number of empirical data points escalates as sampling resolution increases in these multidimensional systems. However, as figure 5 illustrates, sampling at a low, more-clinically-feasible resolution and interpolating using simple linear methods produced small end-point errors ranging from 0 to 2 cm through most of the workspace with only slightly higher errors at the extremes. Here we limited our interpolation methods to the simplistic method of MATLAB’s linear or cubic spline interpolation (similar results were achieved using both) applied to one degree of freedom at a time. This simplistic interpolation method reflects the upper bound on the resulting errors to highlight the impact of sampling resolution on limb endpoint accuracy. However, these interpolation errors are likely to be further reduced by using more sophisticated interpolation methods that address nonlinear changes with interactions across dimensions.

One should note that the errors reported in figure 5 only reflect interpolation errors. Real FES systems will have additional sources of errors such as measurement errors in the empirical data used to initially build the controller, errors related to non-stationarity of the muscle’s response over time, and even errors in the decoded command signal. Two properties of brain-controlled FES systems can offset these inevitable errors. First, the users will have visual feedback and can modify their command signals to compensate for small errors in real time. The brain-controlled trajectories in figure 6 illustrate this point where slight deviations in the straight-line path are easily corrected for by redirecting the arm back to the intended target. With further practice, consistent deviations in the intended movement will likely lead to stable feedforward corrections and straighter movements as the user updates its internal model of how the arm behaves in response to the given command [35].

Second, an initial lookup table could be built with sparse empirical data and interpolation to give the user some function quickly. Then additional empirical passive/active torque data could be collected with an exoskeleton, and the table refined further on subsequent clinical visits to gradually replace less accurate interpolated values. Additionally, further refinements could be made during at-home use by periodically collecting data on where the limb actually goes in response to the different stimulation settings using position or joint angle sensors attached to the limb or newer image processing algorithms that can extract limb kinematics from video data. Or more simply, an at-home calibration routine could be periodically employed without sensors or video processing simply by having the user touch a series of target locations throughout their workspace and monitoring what desired limb configuration they had to provide to the controller to reach that location. Any mismatches between the desired position command and actual resulting position could be used to adjust the current lookup tables to better match how the arm is currently performing. This at-home recalibration process could address slow non-stationarities such as if frequently stimulated muscles become stronger with FES use over time.

More rapid non-stationarities in the arm’s response to stimulation are likely to come from fatigue during extended periods of use. Here again, visual feedback during use will enable the user to make some corrections in real time simply by redirecting their command signal to compensate for observed errors. However, there are limits to this correction process if, for example, some muscles become too fatigued to achieve the desired limb configuration at any stimulation level. The methodology laid out in this paper serves to minimize errors due to fatigue in three ways. First the ‘minimum norm’ cost function ensures work is distributed across redundant muscle thus preventing reliance on any one muscle for force production. Second, the added stiffness dimension in the controller makes it easy to conserve muscle strength by reducing the degree of cocontraction when the stiffness needs are low. Finally, we showed in this paper how to build more fatigue-resistant lookup tables when there are known differences in the fatigue rates of different muscles.

Both the standard and fatigue-resistant lookup tables generate stimulation settings that correctly achieve the desired limb configurations initially. However, the process described in section 2.5 shows how one can artificially scale terms in the system of equation to skew how the resulting stimulations distribute work across redundant muscle. This skewing process can be used to ensure the more easily fatigued muscles are stimulated less thus slowing the effects of fatigue.

For this study, we used a simplified constant fatigue rate in only one or two muscles at a time to demonstrate the process of redistributing work across muscles. In practice, most muscles will fatigue to some extent over time, and how fast each muscle fatigues will vary based on the use and rest cycles of a given task. Additionally, the rate of fatigue is likely not constant across different stimulation levels so scaling down one easily-fatigued muscle may increase stimulation of a different muscle, possibly putting that muscle into a more easily fatigued state. Therefore, this method should be used with caution and only implemented when fatigue in specific muscles is clearly causing a problem, and the remaining muscles have the capacity to compensate.

The process described here for redistributing the balance of stimulation away from certain muscles has more applications than just how we used it here to make the lookup tables more fatigue resistant. One might want to reduce stimulation on electrode channels where stimulation causes pain or produces excessively large artifacts on the cortical recordings. Another example might be if different sets of channels are connected to different power supplies and one power supply is getting drained more quickly than the other. One could use the process described in section 2.5 to arbitrarily shift the balance of work away from the channels that are draining their battery pack more quickly.

The lookup table method used here is an advancement of the previous lookup table control method used by the first spinal cord injured human to control FES-activated reaching using commands directly decoded from intracortical microelectrodes in the motor cortex [19]. That first lookup table controller was based on sparse trial-and-error data collected by an engineer with over 20 years of FES experience. The resulting lookup tables did not allow for variations in the degree of stiffness, or account for complex interactions between joints, and most importantly, that trial-and-error calibration process is not easily transferable to other institutions that do not have such experienced engineers available. Here we lay out an *objective* process based on simple straightforward mathematical principles that could allow anyone to generate optimized lookup tables using empirically collected passive and active joint torque data.

The lookup tables that result from this calibration process define the stimulations to apply that would place the limb in the specified configuration and desired stiffness level. To use this table to make arbitrary movement paths, one would calculate the next desired point in a path at each time loop (e.g. by decoding it from the brain in the BMI-controlled case) and ‘look-up’ the stimulations to apply to achieve the updated desired limb state similar to the process used in [19]. The process is well suited for use with continuous decoding of position or velocity commands in either Cartesian or joint-angle space. Velocity commands are typically integrated over time anyway to get the next desired position [36], and conversion between Cartesian and joint-angle space can be easily calculated. An appropriate stiffness could be automatically calculated from the velocity as in [28] and/or decoded as an independent command signal to give the user volitional control of the stiffness of the limb.

In addition to being able to generate arbitrary movement paths by using the lookup table with a continuously updating command signal, this lookup table method lends itself well to other types of BCI decoding systems. For example, it could be employed with a ‘goal decoder’ that determines the final position the user wants their arm to be in but not the path it should use to get there. In that case, once the desired goal position has been identified, one could simply look up and apply the stimulation levels needed to achieve the final desired position. However, if the distance the arm needs to travel is relatively large, such a large sudden change in muscle stimulation levels could send the arm flying, and the momentum could cause the arm to overshoot and oscillate around the target before settling down at the goal position. In that case, one might want to make a more gradual transition from the current stimulation parameters to the new goal parameters and incorporate some automated increase in stiffness as the arm decelerates to approach the goal to further help stabilize the arm.

Note, the simple controller design described here is a ‘quasi-static’ control method where the inertial properties of the arm are not taken into account. Quasi-static control methods have been shown to work well for low to moderate speed movements where the dynamic effects of the arm are relatively small [12, 18] although errors are likely to increase at higher speeds. This simplified system does not incorporate any feedback control in the controller itself, and therefore does not require any embedded sensors to track limb motion. Instead, it is reliant on the visual feedback of the user to correct for any movement errors, and it sets the stage for further testing to see how much complexity is really needed in the FES controller versus how much of the error correction process should be left to the adaptive brain.

It is our goal that the methodologies described here will lead to improvements in the independence and quality of life of paralyzed individuals by making FES a more practical and clinically deployable option for restoring arm function after spinal cord injury.

## Data Availability

The model arm used in this study is freely available at https://simtk.org/projects/das. The data that support the findings of this study are openly available at the following URL/DOI: https://zenodo.org/records/15151235.
